# Diffusion tensor distribution imaging of an *in vivo* mouse brain at ultrahigh magnetic field by spatiotemporal encoding

**DOI:** 10.1002/nbm.4355

**Published:** 2020-08-19

**Authors:** Maxime Yon, João P. de Almeida Martins, Qingjia Bao, Matthew D. Budde, Lucio Frydman, Daniel Topgaard

**Affiliations:** ^1^ Department of Chemical and Biological Physics Weizmann Institute Rehovot Israel; ^2^ Division of Physical Chemistry, Department of Chemistry Lund University Lund Sweden; ^3^ Random Walk Imaging AB Lund Sweden; ^4^ Medical College of Wisconsin Wauwatosa Wisconsin USA

**Keywords:** acquisition, diffusion, diffusion MR sequences, high‐order diffusion MR

## Abstract

Diffusion tensor distribution (DTD) imaging builds on principles from diffusion, solid‐state and low‐field NMR spectroscopies, to quantify the contents of heterogeneous voxels as nonparametric distributions, with tensor “size”, “shape” and orientation having direct relations to corresponding microstructural properties of biological tissues. The approach requires the acquisition of multiple images as a function of the magnitude, shape and direction of the diffusion‐encoding gradients, leading to long acquisition times unless fast image read‐out techniques like EPI are employed. While in previous *in vivo* human brain studies performed at 3 T this proved a viable option, porting these measurements to very high magnetic fields and/or to heterogeneous organs induces *B*_0_‐ and *B*_1_‐inhomogeneity artifacts that challenge the limits of EPI. To overcome such challenges, we demonstrate here that high spatial resolution DTD of mouse brain can be carried out at 15.2 T with a surface‐cryoprobe, by relying on SPatiotemporal ENcoding (SPEN) imaging sequences. These new acquisition and data‐processing protocols are demonstrated with measurements on *in vivo* mouse brain, and validated with synthetic phantoms designed to mimic the diffusion properties of white matter, gray matter and cerebrospinal fluid. While still in need of full extensions to 3D mappings and of scanning additional animals to extract more general physiological conclusions, this work represents another step towards the model‐free, noninvasive *in vivo* characterization of tissue microstructure and heterogeneity in animal models, at ≈0.1 mm resolutions.

## Graphical abstract

Diffusion tensor distribution (DTD) imaging allows to quantify the heterogeneity of tissues in terms of local “sizes”, “shapes” and orientations, which are in turn directly related to the microstructural properties of biological tissues. Here, a high‐definition DTD analysis of a mouse brain is presented, carried out at 15.2 T with a surface‐cryoprobe and relying on a SPatiotemporal ENcoding (SPEN) imaging sequence. These results enabled the model‐free, noninvasive characterization of tissue microstructure and heterogeneity in live animals, at ≈0.1 mm resolutions.

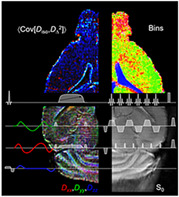

**Abbreviations used**
ADC: apparent diffusion coefficient
Aq: sylvius aqueduct
CB: cerebellum
cc: corpus callosum
CSF: cerebrospinal fluid
D3V: dorsal third ventricle
DTD: diffusion tensor distribution
DTI: diffusion tensor imaging
FA: fractional anisotropy
FE: fractional eccentricity
fi: fimbria
Gl: granular insular cortex
GM: gray matter
GrO: granular layer
LV: lateral ventricles
MA: microscopic anisotropy index
MD: mean diffusivity
SE‐EPI: spin echo‐echo planar imaging
SPEN: SPatiotemporal ENcoding
WM: white matter
μFA: microscopic fractional anisotropy

## 1 INTRODUCTION

The translational motion of water within brain tissues is restricted by biomembranes forming the outer borders of individual cells as well as of intracellular organelles such as the mitochondria, the endoplasmic reticulum and the cell nucleus. Membranes also constitute the multilayer stacks defining myelin sheaths. The micrometer‐scale organization of these biomembranes imprints itself on the diffusion properties of the water in the tissues,^1^, ^2^ which can be noninvasively assessed with magnetic resonance imaging (MRI)^3^ via diffusion‐weighting gradients. Observables from diffusion‐weighted MRI are conventionally expressed in terms of quantitative metrics such as the apparent diffusion coefficient (ADC),^4^, ^5^, ^6^ mean diffusivity (MD) and fractional anisotropy (FA).^7^ This metric calls for the use of diffusion tensor imaging (DTI)^8^ modalities, where the water diffusivity properties are approximated as a rank‐two tensor for each imaged voxel.^9^ DTI has been widely used to study white matter (WM) diseases,^10^ and to track WM pathways in both animal models and humans.^11^, ^12^, ^13^ The orders of magnitude that separate the length scales of water diffusivity (μm) from the imaging resolution (mm) probed by MRI provide these approaches with an intrinsic sensitivity advantage when trying to examine microstructures noninvasively. However, they also introduce ambiguities in the interpretation of conventional DTI data, particularly for heterogeneous voxels containing multiple tissue types^2^, ^14^ or for WM tracts with complex fiber configurations.^15^ Increasing the spatial resolution partially mitigates these heterogeneity effects^16^ but remains challenging, especially *in vivo*, where signal‐to‐noise ratio (SNR) considerations and restrictions on measurement time put practical limits on the achievable imaging resolution.^17^, ^18^, ^19^, ^20^

Diffusion tensor distribution (DTD) addresses this challenge by describing the contents of a voxel comprising multiple tissue environments, each possessing a distinct diffusion tensor **D**, in terms of a *P*(**D**) probability function.^21^ This *P*(**D**) is related to the diffusion‐encoded signal *S*(**b**) imaged by DTI as the integral transform

(1) $$S\left(b\right)=S_{0}\int P\left(D\right)\exp\left(-b:D\right)dD,$$

where *S*_0_ is the initial signal amplitude, **b** is the diffusion‐encoding second‐rank tensor generated by the diffusion‐measuring gradients,^22^ and the colon represents the generalized scalar product **b** : **D** = ∑_*i*_∑_*j*_*b*_*ij*_*D*_*ij*_. Following the treatments in Jian et al^21^ and Anderson^23^ we constrain the integral in Equation 1 to the space of axisymmetric and semipositive‐definite diffusion tensors; these in turn are fully characterized by four independent parameters: the isotropic diffusivity *D*_iso_, the normalized anisotropy *D*_Δ_,^24^ and the polar and azimuthal angles *θ* and *φ*, giving the orientation of the tensor in the lab frame of reference. The two former parameters are related to the axial and radial diffusivities, *D*_||_ and *D*_⊥_, via *D*_iso_ = (*D*_||_ + 2*D*_⊥_)/3 and *D*_Δ_ = (*D*_||_– *D*_⊥_)/3*D*_iso_. When visualizing the tensors as ellipsoid or superquadric glyphs, the values of *D*_iso_ and *D*_Δ_ are directly related to the “sizes” and “shapes” of the displayed objects,^25^ which are properties inherited from the corresponding diffusion propagators and underlying micrometer‐scale tissue structure.^26^ Access to the DTD thus allows the discrimination and structural characterization of various sub‐voxel tissue environments, based on their diffusion properties. Moreover, multiple statistical descriptors carrying different information can be derived from the DTD. The mean “size” E[*D*_iso_] is identical to the classical MD. The mean squared “shape” E[*D*_Δ_^2^] provides similar information as previously introduced anisotropy measures such as the microscopic anisotropy index (MA),^27^ the fractional eccentricity (FE)^28^ and the microscopic fractional anisotropy (μFA).^29^, ^30^ All these pure “shape” measures are conceptually different from the traditional FA,^7^ which convolves the fundamentally different properties of “shape” and orientational order into a single scalar metric.^29^, ^31^ The DTD analysis also provides the (co)variances Var[*D*_iso_], Var[*D*_Δ_^2^] and Cov[*D*_iso_,*D*_Δ_^2^], which report on various aspects of intra‐voxel heterogeneity.^32^ In particular, the Var[*D*_iso_] parameter shows elevated values in voxels containing distinct intra‐ and extracellular water populations,^29^ and has been shown to correlate with cell density heterogeneity in brain tumors.^33^

Recently, acquisition and analysis protocols allowing unconstrained inversion of Equation 1 and subsequent retrieval of the DTD have been introduced.^32^, ^34^, ^35^ Key to these approaches is the combined use of multidimensional diffusion MRI methods,^36^ in which the signal is encoded according to the principles of solid‐state NMR experiments for correlating isotropic and anisotropic chemical shifts,^37^, ^38^ together with data inversion strategies from low‐field NMR incorporating uncertainty estimation of the metrics derived from the distributions arising from Equation 1.^39^ In practice, the measurements involve acquiring data with *b* **‐**tensors of varying magnitude *b*, normalized anisotropy *b*_∆_ ^24^ defined by the axial and radial eigenvalues, *b*_||_ and *b*_⊥_, according to *b*_∆_ = (*b*_||_‐ *b*_⊥_)/*b*, and orientations (Θ, Φ), in order to establish correlations across the (*D*_iso_, *D*_Δ_, *θ*, *φ*) dimensions of the DTD space. Each tensor is defined by a conventional *b*‐value and normalized anisotropy *b*_∆_ with *b* = *b*_||_ + 2*b*_⊥_ and *b*_∆_ = (*b*_||_‐ *b*_⊥_) /*b.* The “shape” of the diffusion‐encoding tensor is characterized by *b*_∆_ with special values −1/2, 0 and +1 for planar, spherical and linear diffusion encoding, respectively.^24^ While acquiring data with multiple tensorial **b**‐values provides the increased specificity needed to characterize the heterogeneities associated with diffusivity properties, it also results in long acquisition times; dealing with the latter leads in turn to reduced spatial resolution and/or limited sensitivity. Hence, in order to be compatible with *in vivo* acquisition times, DTD imaging is usually performed using fast acquisition schemes such as spin echo‐echo planar imaging (SE‐EPI).^16^ In murine models, where the sample volume is small, sensitivity usually remains the limiting factor for endowing these diffusion MRI measurements with high spatial resolution. Cryo‐coils^40^ and very high magnetic fields^41^ can be used to alleviate this limitation; however, increasing the magnetic field *B*_0_ also enhances the susceptibility‐induced magnetic field inhomogeneities,^41^ leading to image distortions in SE‐EPI. High magnetic fields are also associated with shortened transverse relaxation times *T*_2_, demanding, in turn, the application of stronger magnetic field gradients, which increase eddy current artifacts. While SE‐EPI can partially overcome some of these drawbacks by relying on segmented acquisitions that yield improved resolution and immunity to magnetic field inhomogeneities,^42^ these approaches are notably sensitive to motional and instrumental artifacts.

SPatiotemporal ENcoding (SPEN) provides a single‐shot acquisition module with potentially higher resilience to magnetic field inhomogeneities and to eddy current artifacts than SE‐EPI.^43^, ^44^, ^45^ Indeed, SPEN can be implemented in a fully *T*_2_*‐refocused manner, where field inhomogeneities are compensated throughout the acquisition instead of at a single echoing time.^44^ In addition, SPEN's bandwidth along the blipped dimension—the more artifact‐prone in EPI—is defined at the excitation stage by a chirp pulse and can be set at arbitrary values; this can further decrease sensitivity to magnetic field inhomogeneity, even if at the cost of SNR. Moreover, SPEN records its images directly in real space along the low‐bandwidth dimension; since each signal yields a direct low‐resolution image, this facilitates motion correction and data interleaving between shots, leading to final results that can be freed from motion artifacts in multi‐shot or signal‐averaged acquisitions.^46^, ^47^, ^48^ SPEN's use of a chirped pulse applied in conjunction with an encoding gradient also induces a spatial selectivity, which permits zooming without folding along the low‐bandwidth (blipped) dimension.^49^ Finally, as the offset between even and odd echoes that leads to ghost artifacts in EPI can be corrected in SPEN *a posteriori* via a referenceless method,^46^, ^47^, ^50^ this spares the need for the longer acquisition schemes used in EPI to correct this artifact with double sampling of the readout lines.^51^

In this work, we show that DTD imaging can be performed on *in vivo* mouse brains at ≈100 μm in‐plane spatial resolutions (in this case, with a 700 μm slice thickness) by combining the SNR advantages provided by high magnetic fields (15.2 T) and surface‐cryoprobes, with SPEN methods capable of mitigating the effects of *B*_0_‐ and *B*_1_‐inhomogeneities. The microstructural results provided by the ensuing imaging approach are analyzed, and further understood by ancillary measurements on three synthetic phantoms emulating the diffusion properties of WM, gray matter (GM) and cerebrospinal fluid (CSF).

## 2 MATERIALS AND METHODS

### 2.1 Animals

Measurements were performed on healthy adult female C57BL/6 mice aged ~6 months (*n* = 2, Envigo, Jerusalem). The Institutional Animal Care and Use Committee of the Weizmann Institute of Science, which is fully accredited by the AAALAC, the US NIH Office of Laboratory Animal Welfare, and the Israel Ministry of Health, approved all experiments. The animals were housed in cages in a 12‐hour night/12‐hour daylight cycle, with water and food available ad libitum. Mice were anesthetized with ~3% isoflurane and kept under anesthesia throughout the entire scanning session using ~1.5% isoflurane mixed with 20%/80% oxygen/nitrogen. The animals were kept warm during the experiment by using a water‐heated blanket (positioned under the animals) and setting the external temperature of the cryo‐coil to 37°C. Only respiration was monitored throughout the course of the experiment via a pressure sensor (SA‐II, Stony Brook, NY, USA) and maintained at 30‐60 breaths per minute.

### 2.2 Phantoms

Validation of the acquisition and analysis pipeline was performed by measurements on three phantoms consisting of 15 mm Eppendorf tubes containing either a lyotropic liquid crystal, *n*‐dodecane (Sigma‐Aldrich, Israel), or tap water. The phantoms were designed to give diffusion properties similar to the main components of healthy living brains: the liquid crystal phantom captures anisotropic diffusion within WM, the dodecane phantom mimics slow isotropic diffusion in the GM, and the water phantom approximates the fast isotropic diffusion of CSF. The liquid crystal is given the nickname “hex” for its reverse hexagonal phase structure, and was prepared according to the protocol described in detail by Nilsson et al^52^ using 41.94 wt% H_2_O of Milli‐Q quality, 44.12 wt% of the detergent sodium 1,4‐bis(2‐ethylhexoxy)‐1,4‐dioxobutane‐2‐sulfonate (Sigma‐Aldrich, Sweden), and 13.94 wt% of the hydrocarbon 2,2,4‐trimethylpentane (Sigma‐Aldrich, Sweden).

### 2.3 Image acquisition

**FIGURE 1:**
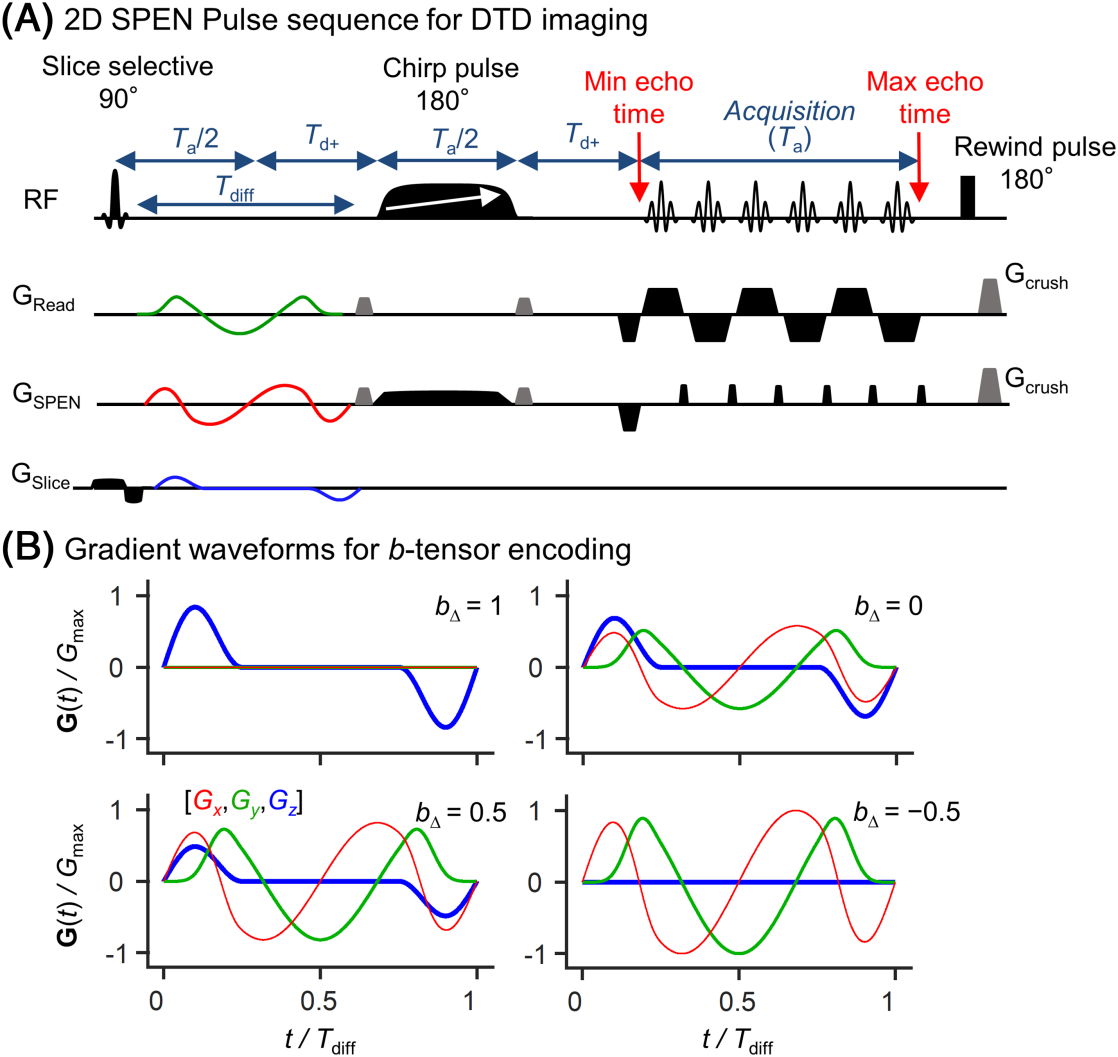
Experimental protocol assayed here for diffusion tensor distribution (DTD) imaging at high spatial resolution. (A) Pulse sequence combining tensor‐valued diffusion encoding with a 2D SPatiotemporal ENcoding (SPEN) acquisition scheme. Diffusion encoding is performed by a set of continuous, self‐refocused gradient waveforms, with duration *T*_diff_, allowing for separate control over the magnitude *b*, normalized anisotropy *b*_∆_ and orientation (Θ, Φ) of the diffusion‐encoding tensor **b**.^34^ The pulse sequence timing is illustrated under conditions that fulfill SPEN's full refocusing condition; to this effect, an additional delay (*T*_d+_) was introduced, if/when the length of the diffusion‐encoding gradient waveform exceeded *T*_a/2_. (B) Set of gradient waveforms used in this work. Each waveform was designed to give a distinct value of *b*_∆_, as per manipulations of the three orthogonal (color‐coded) gradients

All experiments were performed on a horizontal Biospec 15.2 T USR preclinical MRI scanner with an Avance IIIHD console and a B‐GA 6S‐100 three‐axis gradient system with a 60 mm inner diameter, capable of delivering a gradient strength of 1000 mT/m and associated with an integrated first to third order shim set. Data were acquired with a surface ^1^H quadrature transmit/receive cryoprobe coil with an inner diameter of 20 mm. *In vivo* measurements were performed without respiratory triggering, but using ear and teeth bars to fix the mouse's head. Prior to all acquisitions, the *B*_0_ homogeneity within the field of view (FoV) was optimized using the field map method^53^, ^54^, ^55^ and the third‐order shims available. For the *in vivo* experiments, the linewidth at 50% obtained within the targeted FoV by a PRESS sequence was ≈ 60 Hz. Two saturation bands were used to remove the intense signal at the back of the brain and allow better phase adjustment during the SPEN processing. Fat suppression was not used. Additional hardware and imaging parameters used in the *in vivo* and phantom data acquisitions are reported in Table 1. SPEN data were collected with the sequence shown in Figure 1, which contains a refocusing chirp pulse whose length is half of the acquisition time *T*_a_ in order to maintain the condition of full *T*_2_*‐refocusing.^44^ The rasterized image‐space acquisition inherent to the spatiotemporal encoding induces a gradually increasing echo time from 29 to 52 ms, which in turn leads to a nonuniform *T*_2_‐weighting along the SPEN dimension. The initial encoding and its subsequent step‐by‐step refocusing by the acquisition gradients also leads to a variation in the effective *b*‐values along the spatiotemporal encoding axis.^56^ The spatially incremented echo times and progressive diffusion weighting along the SPEN dimension may lead to a gradual skewing of the estimated DTDs towards components with long *T*_2_ and slow diffusion. On the other hand, the fact that the diffusion gradient waveforms are fully refocused before the application of the SPEN gradients means that the diffusion weighting from the dedicated diffusion encoding and the SPEN imaging gradients can be separated into two independent *b*‐tensor factors. The latter span relatively small values in the 15‐250 s/mm^2^ range, which, since constant throughout the DTD waveform variations, could be factored out in the final analysis.

**TABLE 1:** Main parameters of the DTD SPEN acquisitions

Total acquisition time	2 h 25 min
Echo time	59 +/− 11.5 ms
Repetition time	1 s
Interleaved segments	3
Number of scans	8
Field of view	13 × 17 × 0.7 mm^3^
Matrix size	180 × 174 × 1
Readout regridded points (effective)	132
Spatial resolution	99 × 98 × 700 μm^3^
Readout bandwidth	455 kHz
Second dimension bandwidth	10.450 kHz
Chirp duration	11.5 ms
Number of images	364
Number of *b*_0_ images	4 (one per **b** shape)
Diffusion waveform duration	18 ms
*b‐*values	0.042, 0.173, 0.388, 1.080, 2.116 and 3.499 x 10^9^ s/m^2^
*b*_Δ_	−0.5, 0, 0.5 and 1
Number of *b*‐tensor directions	15
Linewidth at 50%	60 Hz
Press shim FOV	5 × 8 × 1 mm^3^
SNR of the *b*_0_ image	26

The SPEN readout was performed during the full echo train acquisition, including the gradient ramps. This induced a nonuniform sampling of the *k*‐space points along the readout direction, which was corrected by regridding^57^ prior to Fourier transform. This operation was taken into account when determining the final spatial resolution, which was calculated based on the evenly spaced *k*‐space points obtained after regridding. Images along the SPEN dimension were reconstructed as described elsewhere^46^, ^47^, ^48^; the main elements of this reconstruction are: a phase correction between even and odd segments, a phase correction in between segments which also induce a rigid motion correction, and a super‐resolution operation. The SPEN sequence and SPEN reconstruction pipeline used in this study are available at https://www.weizmann.ac.il/chemphys/Frydman_group/software.

Diffusion‐encoded images were collected for a full mouse brain coronal slice, with an in‐plane spatial resolution of 100 μm and a slice thickness of 700 μm. A coronal orientation was chosen so as to cover the largest spatial dimensions of the mouse brain entirely. The choice of this challenging orientation, less common than the axial orientation usually used in EPI‐based DWI studies, is possible due to the increased robustness of the SPEN acquisition to *B*_0_ inhomogeneities, and of its ability to suitably scan its spatial axis while avoiding signal folding. The repetition time was 1 second, and three segments were interleaved in each image acquisition. The diffusion‐encoding protocol comprised 364 such image acquisitions, decomposed into four *b*_Δ_‐values (−0.5, 0, 0.5 and 1),^24^ 15 directions, and six *b*‐values ranging from 0.042 to 3.5 x 10^9^ s/m^2^. Gradients were calculated using analytical waveforms^58^ derived on the basis of the variable‐angle spinning technique^38^ in solid‐state NMR. The maximum *b*‐value 3.5 x 10^9^ s/m^2^ was reached at 90% of the maximum available gradient strength (1000 mT/m) using a waveform of duration *Τ*_diff_ = 18 ms. Together with a chirp duration *T*_a_/2 = 11.5 ms, this constrained the SPEN echo times to the 47.5 to 70.5 ms range, which was difficult to reduce by increasing segmentation. To mitigate the low SNR arising as a result of long echo times and relatively short *T*_2_s (~ 25 ms for GM at 15.2 T^59^), a set of eight averages per scan was acquired, leading to a total experiment time of 2 hours and 25 minutes. This scanning time, long but still compatible with *in vivo* preclinical investigations, requires the use of fast acquisition schemes such as EPI or SPEN, both of which provide similar acceleration factors. While these choices allowed us to acquire 2D images with high spatial resolution (100 μm in‐plane) using a limited number of interleaved segments (three), the SNR demands of DTD imaging also required us to average eight scans. Coupling these factors to the large number of images required for DTD data inversion motivates our acquisition of just a single slice.

### 2.4 DTD estimation

All the acquired images were denoised using random matrix theory,^60^ with an optimized 3 × 3 bidimensional kernel. The SNRs of the various images were computed prior to any denoising; SNR values were estimated as the mean value of the GM signal divided by the standard deviation of the noise in an area without signal.

For each voxel, DTDs were estimated from the acquired signal via a Monte Carlo inversion^39^, ^61^ of Equation 1, using an algorithm that has been described in detail previously^32^, ^35^ and that is freely available online (https://github.com/daniel-topgaard/md-dmri). The algorithm approximates the DTD as a discrete set of *N* components {*w_n_*, *D*_||__,*n*_, *D*_⊥,*n*_, *θ _n_*, *φ _n_*}_1__≤ *n* ≤ *N*_, where *w_n_* is the weight of component *n.* Uncertainty estimations were performed using bootstrapping with replacement, to generate 96 DTDs consistent with the input signal data. For the purpose of calculating the parameter maps, the DTDs were converted to statistical descriptors quantifying the total weight/initial signal *S*_0_, as well as means E[*x*], variances Var[*x*] and covariances Cov[*x*,*y*] of the various dimensions of the distribution space.^32^ These statistical descriptors were derived for both the entirety and from smaller divisions (“bins”) of the DTD space, and are reported using a (*D*_iso_, *D*_Δ_^2^, *θ*, *φ*) parametrization rather than the original (*D*_||_, *D*_⊥_, *θ*, *φ*) representation: E[*D*_iso_], E[*D*_Δ_^2^], Var[*D*_iso_], Var[*D*_Δ_^2^] and Cov[*D*_iso_,*D*_Δ_^2^]. Here, we defined three bins meant to capture the contributions of WM, GM and CSF: WM, 0.005 < *D*_iso_ / 10^–9^ m^2^s^–1^ < 2 and 0.25 < *D*_Δ_^2^ < 1; GM, 0.005 < *D*_iso_/10^–9^ m^2^s^–1^ < 2 and 0 < *D*_Δ_^2^ < 0.25; CSF, 2 < *D*_iso_/10^–9^ m^2^s^–1^ < 5 and 0 < *D*_Δ_^2^ < 1. The bootstrap ensembles of scalar descriptors were condensed to a set of averages by computing the medians 〈·〉 of the 96 plausible values of *S*_0_, E[*x*], Var[*x*], and Cov[*x*,*y*]. Parameter maps of 〈*S*_0_〉, 〈E[*D*_iso_]〉, 〈E[*D*_Δ_^2^]〉, 〈Var[*D*_iso_]〉, 〈Var[*D*_Δ_^2^]〉 and 〈Cov[*D*_iso_,*D*_Δ_^2^]〉 were then used to visualize the main features of the estimated DTDs.

## 3 RESULTS AND DISCUSSION

**FIGURE 2:**
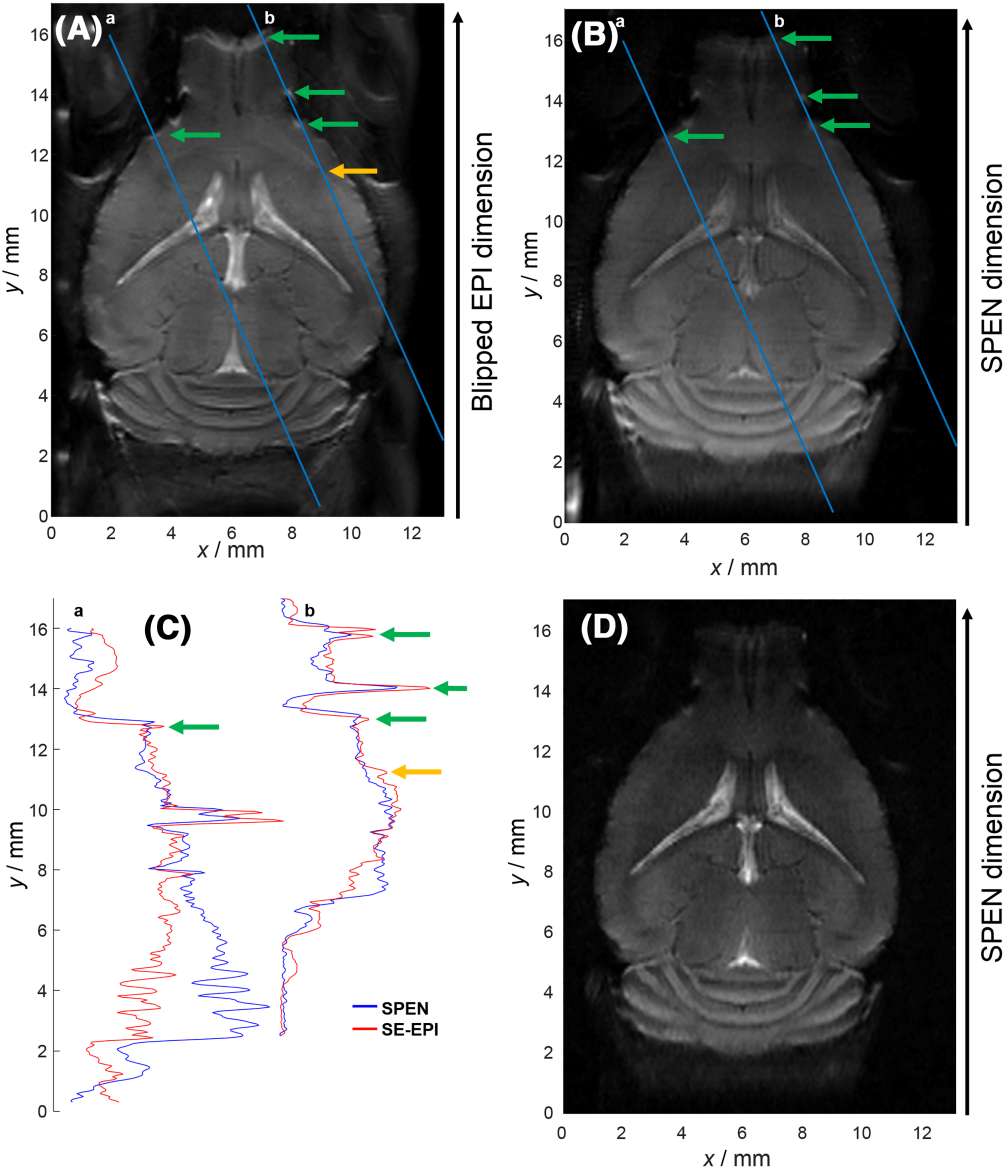
Comparison between images resulting from SE‐EPI and SPEN experiments on an *in vivo* mouse brain coronal slice at 15.2 T using identical shim conditions. All images were acquired with a three‐segment acquisition and had in‐plane resolutions of 100 × 100 µm² and a slice thickness of 700 µm. A, High‐SNR SE‐EPI image recorded with 48 averages leading to an acquisition time of 2 min and 24 seconds. The SE‐EPI sequence had an echo time of 30 ms and an effective bandwidth in the blipped dimension of 7.57 kHz. This image exhibits ghost artifacts like the one shown by the orange arrow and *B*_0_ inhomogeneity artifacts like those shown by the green arrows. B, High‐SNR fully *T*_2_*‐refocused SPEN image recorded with 48 averages leading to an acquisition time of 2 min and 24 seconds. The fully *T*_2_*‐refocused SPEN image was acquired with echo times of 40.5 ± 11.5 ms and an effective SPEN‐dimension bandwidth of 10.45 kHz. The increased bandwidth combined with the full *T*_2_*‐refocusing lead to the higher robustness to *B*_0_ inhomogeneity artifacts compared to the SE‐EPI image, while SPEN’s referenceless reconstruction algorithm^47^ allows one to minimize ghost artifacts. C, Comparison of normalized profiles extracted from the aforementioned SE‐EPI and SPEN images along the blue lines. Arrows indicate the above‐mentioned artefacts. D, *b*_0_ SPEN image acquired with eight averages leading to an acquisition time of 24 seconds and an echo time increased to 59 ± 11.5 ms due to the inclusion of a diffusion delay (*T*_diff_) of 18 ms. This image was denoised using random matrix theory^60^ and a 3 × 3 bidimensional kernel, and is representative of the sets employed in the subsequent DTD calculations

Even when relying on segmented acquisitions combined with animal immobilization using ear and teeth bars, careful optimization of the *B*_0_ homogeneity methods^53^, ^54^, ^55^ and brand new hardware, retrieving full‐brain mouse coronal SE‐EPI images at 15.2 T remains challenging. Figure 2 panel A and B compares one such representative image against a SPEN coronal *in vivo* counterpart, both recorded with high SNR (48 averages), in three segments at a 100 μm in‐plane resolution, under equivalent shimming conditions and similar parameters, except for the echo time and the phase dimension bandwidth. Although the two images show similar anatomical details, contrast differences between CSF and GM are noticeable between the images; these arise mainly due to the longer echo times of SPEN (ranging spatially from 29 to 52 ms) compared with the SE‐EPI acquisition (30 ms). A decay in overall signal intensities along the (antero‐posterior) SPEN dimension is also noticeable; this originates from the progressively stronger *T*_2_ and diffusion weightings induced by the SPEN readout over the course of the image acquisition.^56^ Still, as these weights are constant throughout the various *b‐*encoded acquisitions, their effects factor out from the processed DTD images. On the other hand, the SE‐EPI image evidences a number of ghost artifacts (Figure 2, orange arrow) and of susceptibility‐induced *B*_0_ inhomogeneity artifacts (Figure 2, green arrows). These ghost artifacts could not be eliminated with classical one‐dimensional phase correction via polynomial fitting to reference data scans,^57^ and although they could have been eliminated with a double‐sampling approach,^51^ this would double the EPI acquisition time, which would in turn increase the *B*_0_ inhomogeneity artifacts. It follows that under these good shimming conditions and for the same number of segments, SPEN allows one to obtain these data without ghosts and with much reduced susceptibility‐induced *B*_0_ artifacts, even if subtle stripe‐like artifacts were also evident in the high SNR SPEN image. The absence of significant ghost artifacts in the SPEN image is made possible by its referenceless reconstruction algorithm, including a bidimensional phase correction which minimizes the phase difference between even and odd readout segments, and between shots.^46^, ^62^ This optimization is easier in SPEN due to the direct image space acquisition, which allows the phase optimization to be performed directly in the image space where the phase is smoother than in *k*‐space. The reduction of the *B*_0_ inhomogeneity artifacts is aided by SPEN's increased bandwidth in the second dimension (10.45 vs. 7.57 kHz for SE‐EPI) and by its fully *T*_2_*‐refocused acquisition. The extent of the artifacts reduction is more clearly evidenced in Figure 2C, which compares (in red and blue) normalized 1D profiles extracted from the high SNR SE‐EPI and SPEN images, respectively. Notice in these the reduction in the intensities of the *B*_0_ inhomogeneity artefacts like those evidenced by the green arrows, and the elimination of ghost artefacts of the kind highlighted by the orange arrow. These and other similar artifact attenuations explain our use of SPEN in this kind of study. Figure 2D exhibits one of the SPEN *b*_0_ images within the DTD dataset acquired with the same parameters as the higher SNR SPEN image in panel (B), except for a number of averages reduced to eight and an echo time increased to 59 ± 11.5 ms due to the inclusion of the diffusion delay (*T*_diff_) of 18 ms. This image was denoised using random matrix theory^60^ and a 3 × 3 bidimensional kernel. Notice the difference in contrast between CSF and GM in the two SPEN images arising due to the changing echo times.

**FIGURE 3:**
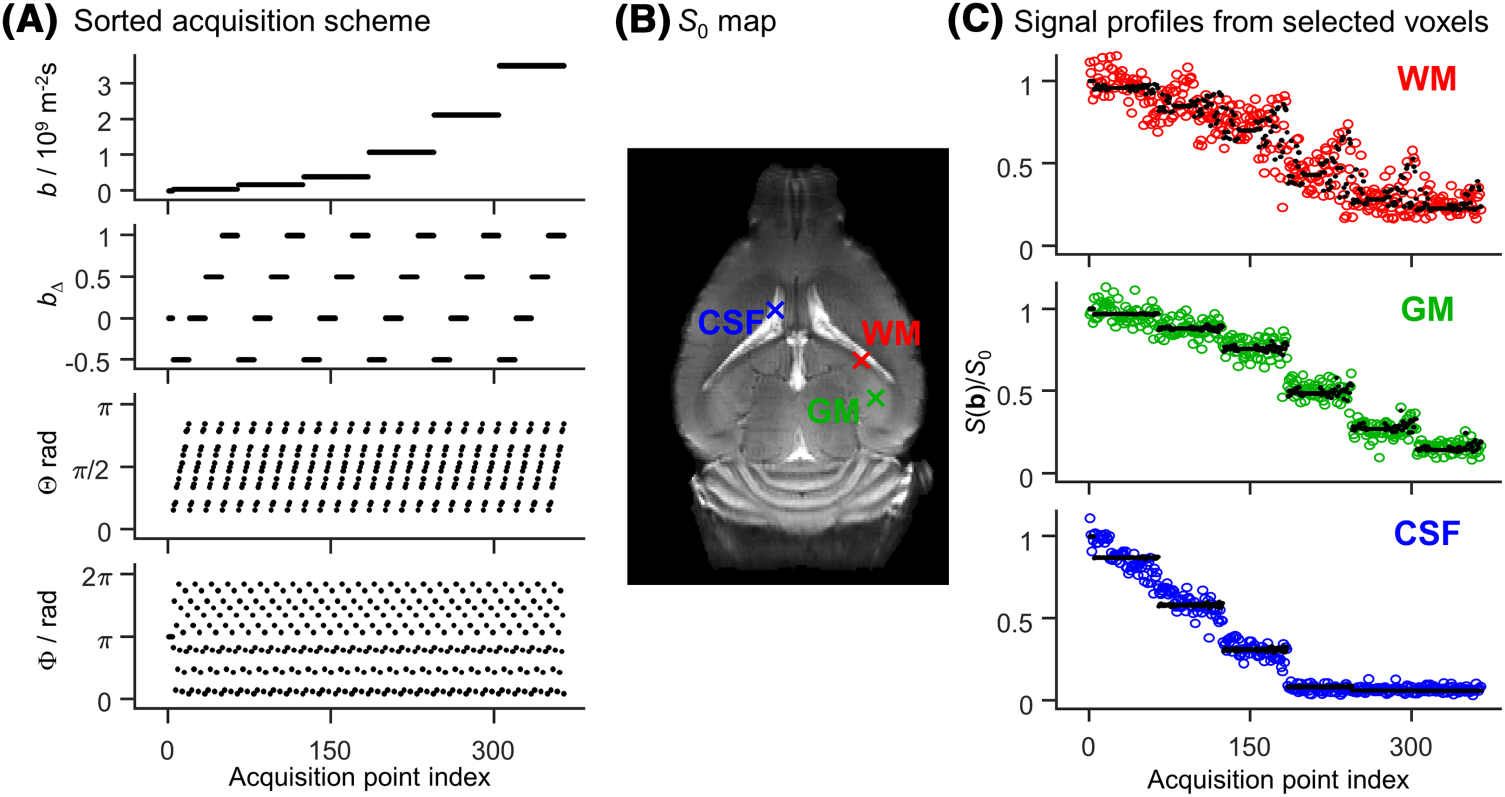
Acquisition protocol and signal data from three representative voxels collected from an *in vivo* mouse brain. (A) Acquisition protocol comprising 364 diffusion encoding tensors **b** ^34^ with six different magnitudes *b*, four different values of normalized anisotropy *b*_Δ_, and 15 orientations (Θ, Φ). The (*b*, *b*_Δ_, Θ, Φ) coordinates are displayed as a function of acquisition point index. (B) Initial signal amplitude *S*_0_, with crosses indicating three voxels containing white matter (WM, red), gray matter (GM, green) and cerebrospinal fluid (CSF, blue). (C) Experimental (colored circles) and fitted (black dots) signal profiles from the three selected voxels. The normalized signals *S*(**b**)/*S*_0_ are sorted according to the acquisition scheme shown in (A)

**FIGURE 4:**
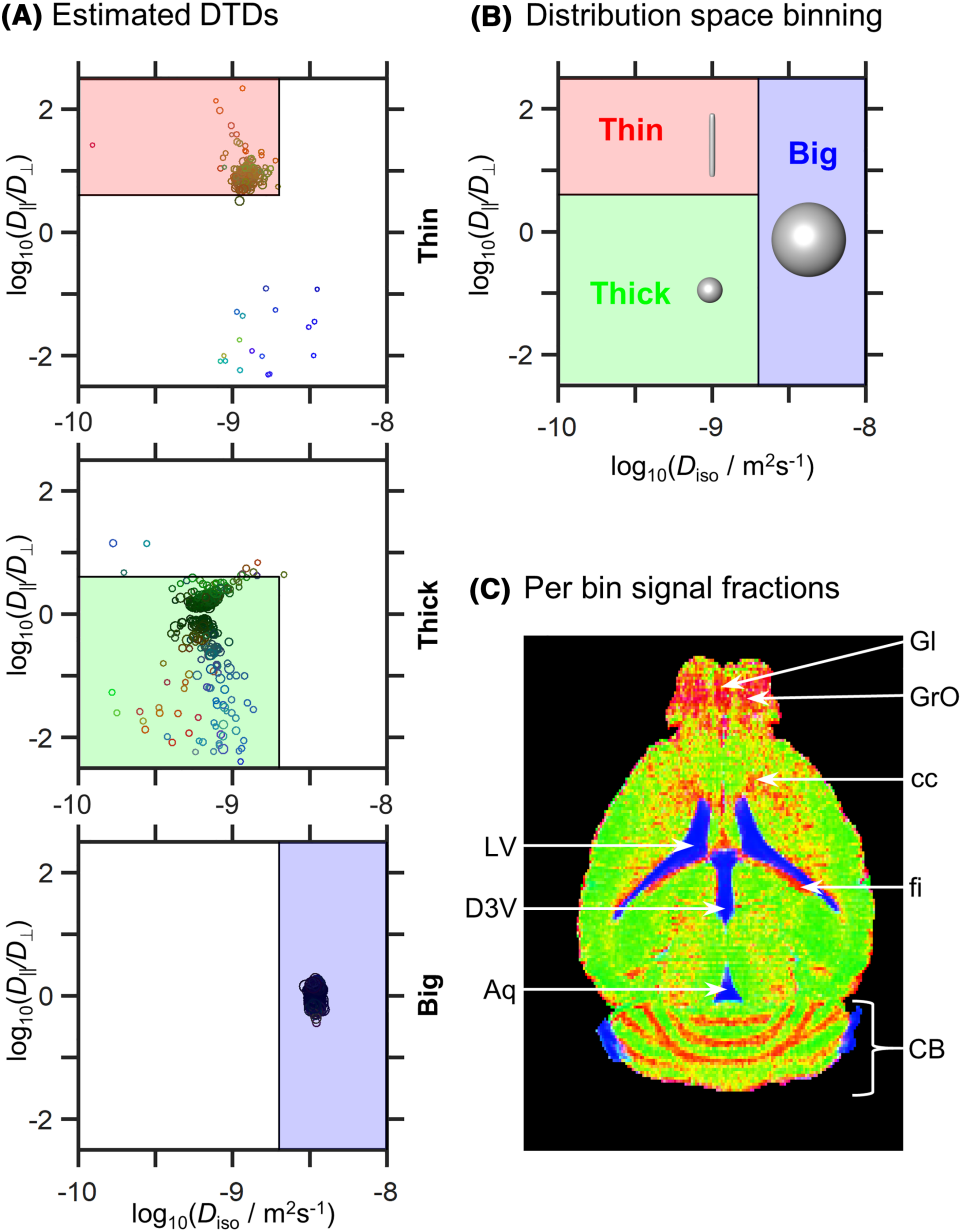
Distribution space and parameter maps derived from subsets (“bins”) of the 4D DTD space. (A) DTDs obtained by nonparametric inversion of the signal data displayed as 2D logarithmic bubble charts of isotropic diffusivities *D*_iso_ and axial‐radial diffusivity ratios *D*_∥_/*D*_⊥_. The component weights and orientations are indicated with the circle areas and colors [R, G, B] = [cos*ø* sin*θ*, sin*ø* sin*θ*, cos*θ*]. (B) Division of the DTD space into three different bins—Thin, Thick and Big—that loosely capture the diffusion features of white matter, gray matter and cerebrospinal fluid, respectively. (C) Signal contributions from the fractional populations in the different bins are visualized as an additive color map ([R, G, B] = [Thin, Thick, Big]). Voxels comprising multiple fractions are visible as a weighted superposition of the three primary colors, eg, equal proportions of Thin and Thick populations yield a yellow voxel. Legend abbreviations^64^: LV, lateral ventricles; D3V, dorsal third ventricle; Aq, sylvius aqueduct; Gl, granular insular cortex of the olfactory bulb; GrO, granular layer of the olfactory bulb; cc, corpus callosum; fi: fimbria; CB, cerebellum

Figure 3 displays some aspects of the DTD experiments and the ensuing SPEN data for three representative voxels containing WM, GM and CSF. For simplicity these results are shown as a function of an acquisition point index (Figure 3A), characterized by independent *b‐*tensor parameters. Each of the voxels yields a distinct signal pattern as a function of this index, highlighting the contrast provided by DTD in relation to the underlying microstructure. The WM voxel is characterized by a signal pattern that is highly influenced by both the anisotropy (*b*_Δ_) and the orientation (Θ, Φ) of the *b*‐tensor, a behavior that is consistent with anisotropic diffusion within and along orientationally ordered axons that dominate WM. CSF and GM voxels yield signal data that are insensitive to both *b*_Δ_ and (Θ, Φ), indicative of isotropic media. Yet the CSF signal differs from GM signals by its sharper decay with increasing *b*‐values, reflecting a higher diffusivity. These various signal patterns can be processed as described above, to retrieve distinct parameters quantifying the main diffusivity features of the underlying microscopic environments. As done in previous studies,^26^, ^32^, ^34^, ^35^ Figure 4A reports these distributions in a logarithmic space based on the diffusion tensor's “size” log_10_(*D*_iso_), and “shape” log_10_(*D*_||_/*D*_⊥_). Within this representation we observe that CSF is characterized by large (log_10_(*D*_iso_/m^2^s^−1^) ≈ −8.5) and isotropic (log_10_(*D*_||_/*D*_⊥_) ≈ 0) diffusion tensors, that WM and GM both present a lower MD compared with CSF (−9.2 < log_10_(*D*_iso_/m^2^s^−1^) < −8.8), and that the DTDs from WM comprise highly anisotropic diffusion components (log_10_(*D*_||_/*D*_⊥_) ≈ 1). While the displayed DTDs capture these gross properties even from a simple visual inspection of the signal patterns, the accuracy of the data inversion process is challenged by the images' limited SNR. Noise scatters the signal profiles (see, for instance, Figure 3C), giving rise to a general broadening of the distributions as well as to the emergence of spurious components that are not biologically plausible; eg, the planar components with log_10_(*D*_||_/*D*_⊥_) ≈ −2 for WM and GM in Figure 4A. A more subtle effect also noticed for the noise, is to bias the inversion algorithm towards finding combinations of anisotropic components, instead of a single isotropic one; this gives rise to the nearly symmetric “butterfly” spread of components centered about the log_10_(*D*_||_/*D*_⊥_) = 0 line in the “Thick” DTD. This effect is analogous to the well‐known positive bias in the anisotropy metrics of conventional DTI at low SNR.^31^ A map of the ratio between the initial signal amplitude *S*_0_ and the square root of the residual sum of squares (RSS), *S*_0_/(RSS)^1/2^, is included in Figure S1 panel A, and provides an assessment of the SNR levels throughout the whole brain images. A second map, describing the ratio between the mean of *S*(*b*,*b*_Δ_ = 0) data points and their standard deviation at the highest *b*‐value (3.499 x 10^9^ s/m^2^), is also presented in Figure S1 panel B, and shows that signal still remains at high *T*_2_ and *b*‐value weightings. SNR‐limited scattering notwithstanding, we find it convenient to divide the experimental DTD SPEN results into bins that we refer to as “Thin”, “Thick” and “Big”, to describe the visual appearance of the corresponding tensor glyphs; this facilitates the separation of signal contributions into components with distinct diffusion properties. Slowly diffusing anisotropic and isotropic components appear in the bins labeled Thin and Thick, respectively, while fast‐diffusing components are captured by the Big bin. Figure 4B shows the boundaries that were assumed for these bins. Figure 4C maps how the sampled signals fractionate into these bins, with the aid of an additive color map display. CSF‐rich areas such as the lateral ventricles (LV), the dorsal third ventricle (D3V) and the sylvius aqueduct (Aq), are all well captured by the Big bin (blue voxels). WM‐rich areas such as (from bottom to top) the WM in the cerebellum (CB, which correspond to the intense red voxels), the fimbria (fi, which correspond to the red voxels below the LV) and the corpus callosum (cc, which correspond to the dispersed red voxels above and in between the LV), appear primarily as the Thin bin. So do the granular layer (GrO) as well as the granular insular cortex (Gl) of the olfactory bulb, which are, respectively, the central and lateral structures of this organ. While literature reports and preliminary data (not shown) validate the main features of high FA in the GrO and GI structures, as reflected in Figure 4C for the olfactory bulb, SNR limitations in this region probably exacerbate spurious components with high anisotropy. This may lead to an overestimation of E[*D*_Δ_^2^], as well as to an overestimation of the diffusion anisotropy and of the Thin bin fractions observed in this region. The separation of Thin and Thick signal populations also identifies WM/GM partial volume effects in the vicinity of the cc (yellow voxels above the ventricles), and in the CB at the edges of the WM areas. The mixture of red and yellow voxels characterizing the cc in Figure 4 is in part reflecting partial volume effects arising mainly due to the relatively large slice thickness chosen (0.7 mm), but is also reinforced by the human brain‐based boundary values chosen for the various bins. Indeed, the mouse cc is intrinsically not as anisotropic as the human one: the conventional FA in cc is ~ 0.5‐0.6 for mouse,^19^ and ≈0.8‐0.9 for human. Hence, values of ≈0.5 fall on the edge between the Thin and Thick bins optimized for human brain segmentations,^63^ and result in the admixing of a yellow contribution to this representation.

**FIGURE 5:**
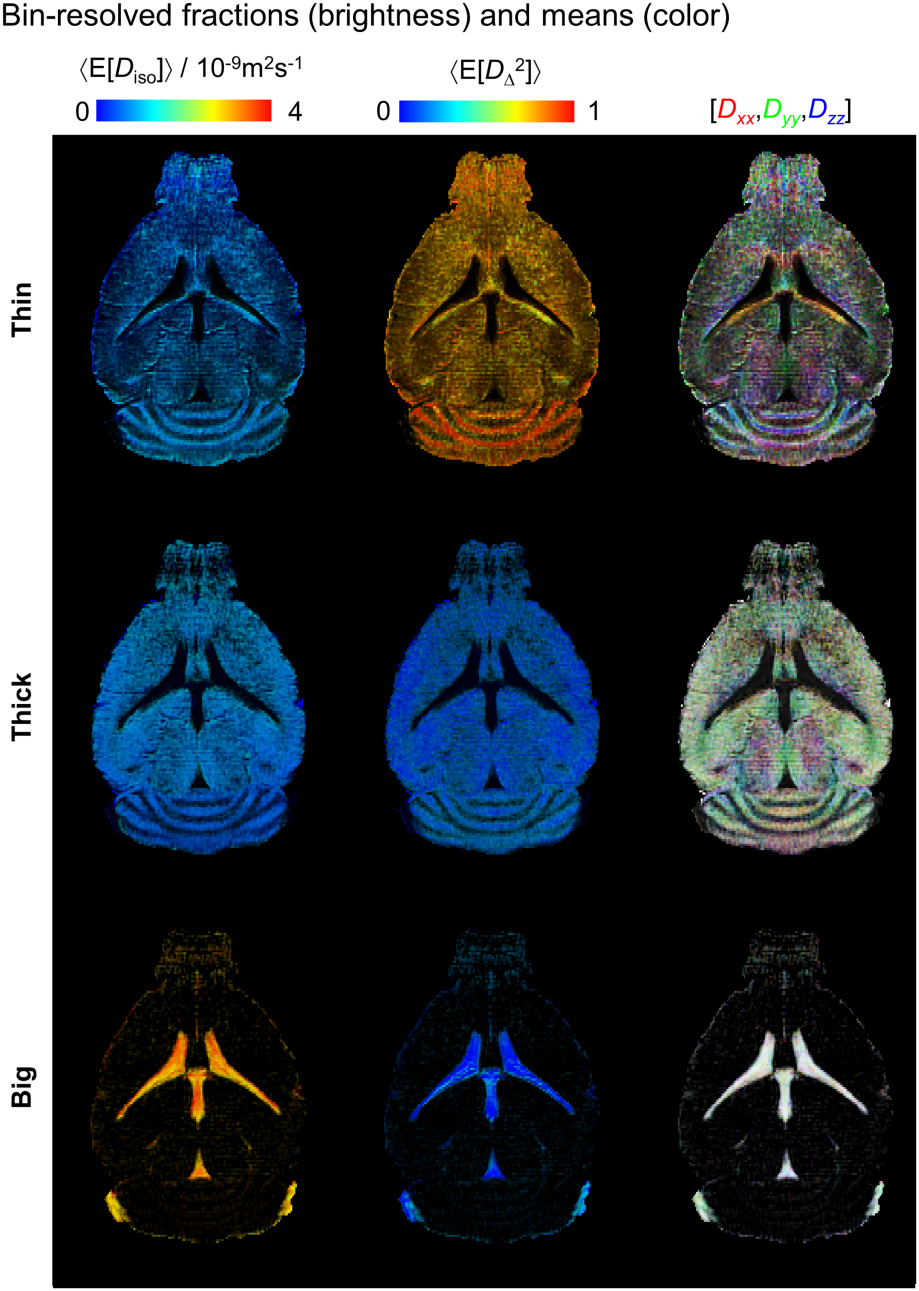
Parameter maps derived from subsets (“bins”) of the 4D DTD space. Per‐bin signal fractions (brightness) and per‐bin average mean values (color) are shown for the isotropic diffusivity 〈E[*D*_iso_]〉, the squared normalized diffusion anisotropy 〈E[*D*_Δ_^2^]〉, and the diffusion tensor orientation 〈E[Orientation]〉. The values of 〈E[*D*_iso_]〉 and 〈E[*D*_Δ_^2^]〉 are indicated by the corresponding linear color scales, while the 〈E[Orientation]〉 maps are color‐coded as [R, G, B] = [*D_*xx*_*,*D_*yy*_*,*D_*zz*_*]/max(*D_*xx*_*,*D_*yy*_*,*D_*zz*_*), where *D_*ii*_* are the *i*‐th diagonal elements of the diffusion tensor as measured in the laboratory frame of reference

**FIGURE 6:**
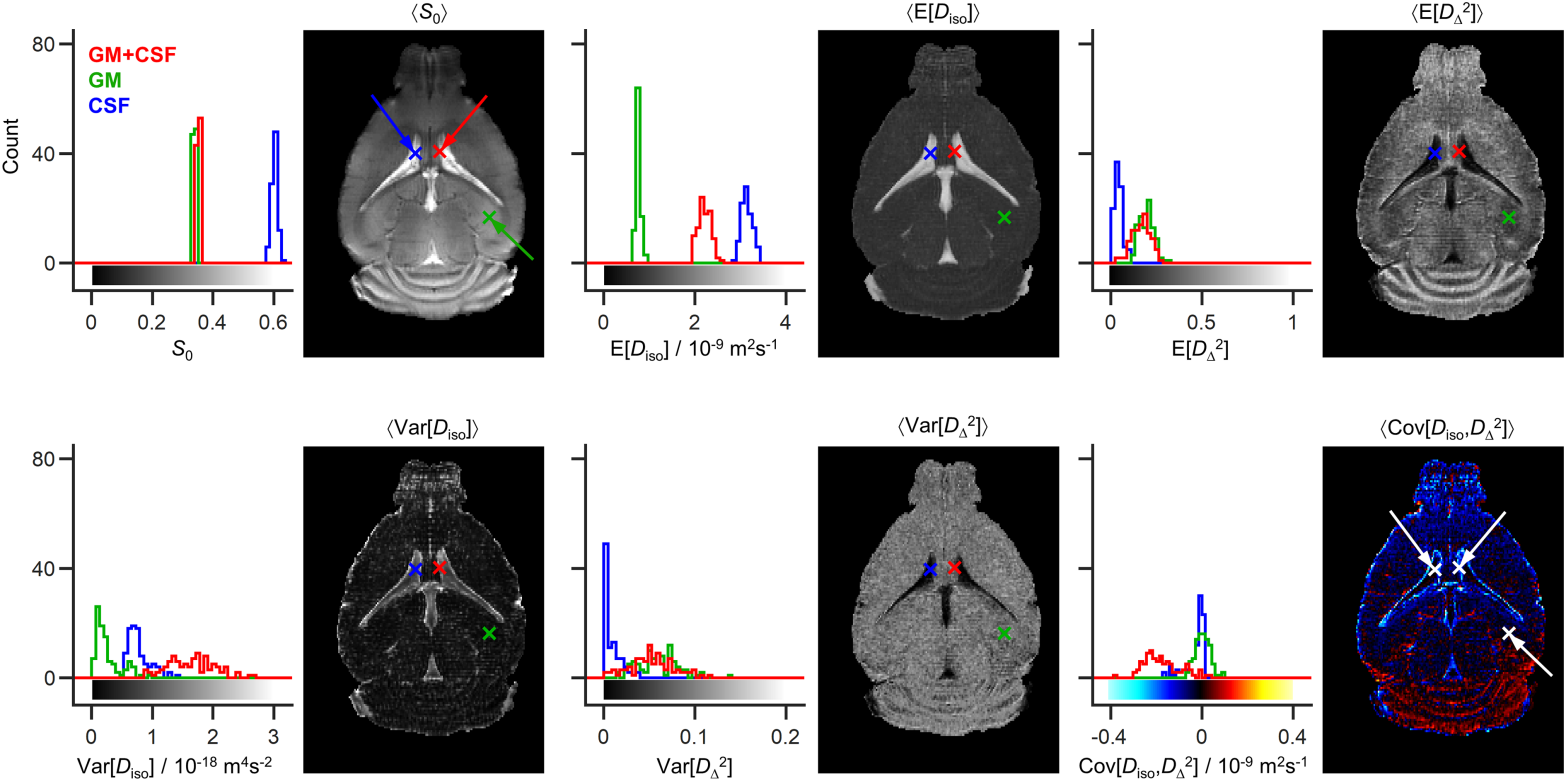
Statistical descriptors derived from the 4D diffusion tensor distributions (DTDs) introduced in Figures 4 and 5. For each voxel, the bootstrapping yields 96 different DTDs consistent with the signal data. The sets of DTDs were converted to sets of the following statistical descriptors: total signal amplitude *S*_0_, mean E[*x*], variance Var[*x*] and covariance Cov[*x*,*y*], for the isotropic diffusivity *D*_iso_ and the squared normalized diffusion anisotropy *D*_Δ_^2^. The parameter maps show 〈*S*_0_〉, 〈E[*x*]〉, 〈Var[*x*]〉 and 〈Cov[*x*,*y*]〉, where the angular brackets indicate averages over these 96 bootstrap realizations. The results from all 96 bootstrap realizations are also shown as histograms for three selected voxels containing gray matter (GM, in green), cerebrospinal fluid (CSF in blue), and a binary mixture of GM and CSF (GM + CSF, in red). The linear color scales of the various maps are indicated by the bars along the horizontal axes of the corresponding histograms

Analyses of the mean parameter maps enable further characterization of the bin‐resolved signal fractions (Figure 5). For example, inspection of the 〈E[Orientation]〉 maps from the Thin bin reveals the alignment direction of the anisotropic tissues found in the CB and the fi. It should be noted that the Thin 〈E[*x*]〉 maps possess a noisy appearance that is characterized by the existence of multiple voxels with low brightness. While such voxels hamper the visual inspection of the Thin maps, their reduced brightness indicates that they have a small contribution to the retrieved DTDs (this is consistent with the overall “green” appearance of the composite map in Figure 4C). For simplifying the analysis of these distributions, the spatially resolved DTDs were converted into 16 individual parameter maps, following the procedure detailed in the Materials and Methods (section 2). Selected parameter maps derived in this manner from the full DTD space are shown in Figure 5. Conventional diffusion protocols can be used to estimate *S*_0_ and E[*D*_iso_], whereas the E[*D*_Δ_^2^], Var[*D*_iso_], Var[*D*_Δ_^2^] and Cov[*D*_iso_,*D*_Δ_^2^] metrics can only be retrieved with tensor‐valued diffusion protocols.^25^, ^28^, ^31^ The E[*D*_iso_] and E[*D*_Δ_^2^] metrics provide measures of MD and diffusion anisotropy, respectively, and can be used to obtain a rough characterization of the spatial distribution of CSF (high E[*D*_iso_], low E[*D*_Δ_^2^]), WM (low E[*D*_iso_], high E[*D*_Δ_^2^]) and GM (low E[*D*_iso_], low E[*D*_Δ_^2^]). It is important to highlight that the E[*D*_Δ_^2^] metric quantifies diffusion anisotropy independently from the underlying degree of orientational order; this is in contrast to common anisotropy metrics such as the FA,^7^ which convolve the fundamentally different properties of diffusion anisotropy and orientational order into a single scalar value.^29^ Notice that, according to the 〈E[*D*_Δ_^2^]〉 map, this mean anisotropy metric is for the most part characterized by small but nonzero values. Whether such small anisotropy reflects the tissue properties of the mouse brain or originates from the bias discussed in the previous paragraph is a point that merits further investigation. Intra‐voxel heterogeneities can be further studied through inspection of (co)variance maps of the type shown in Figure 6. Notice, for instance, how voxels comprising mixtures of CSF and GM (in red) display intermediate values of E[*D*_iso_] and are characterized by elevated Var[*D*_iso_], nonnegligible Var[*D*_Δ_^2^] and negative Cov[*D*_iso_,*D*_Δ_^2^]. The high Var[*D*_iso_] indicates a mixture of slow‐ and fast‐diffusing components,^29^ and the negative Cov[*D*_iso_,*D*_Δ_^2^] suggests that the slower diffusing components possess a higher diffusion anisotropy.^32^ These results are consistent with a linear combination of the diffusion properties measured in pure CSF (〈E[*D*_iso_]〉 ≈ 3.1 x10^–9^ m^2^s^−1^, 〈E[*D*_Δ_^2^]〉 ≈ 0) and GM (〈E[*D*_iso_]〉 ≈ 0.7 x 10^−9^ m^2^s^−1^, 〈E[*D*_Δ_^2^]〉 ≈ 0.2) voxels. The Var[*D*_Δ_^2^] metric has been shown^32^ to correctly identify voxels containing both isotropic and anisotropic diffusion components in a synthetic phantom. The nonzero 〈Var[*D*_Δ_^2^]〉 values may then be interpreted as indicating mixtures of anisotropic and isotropic tissues.

**FIGURE 7:**
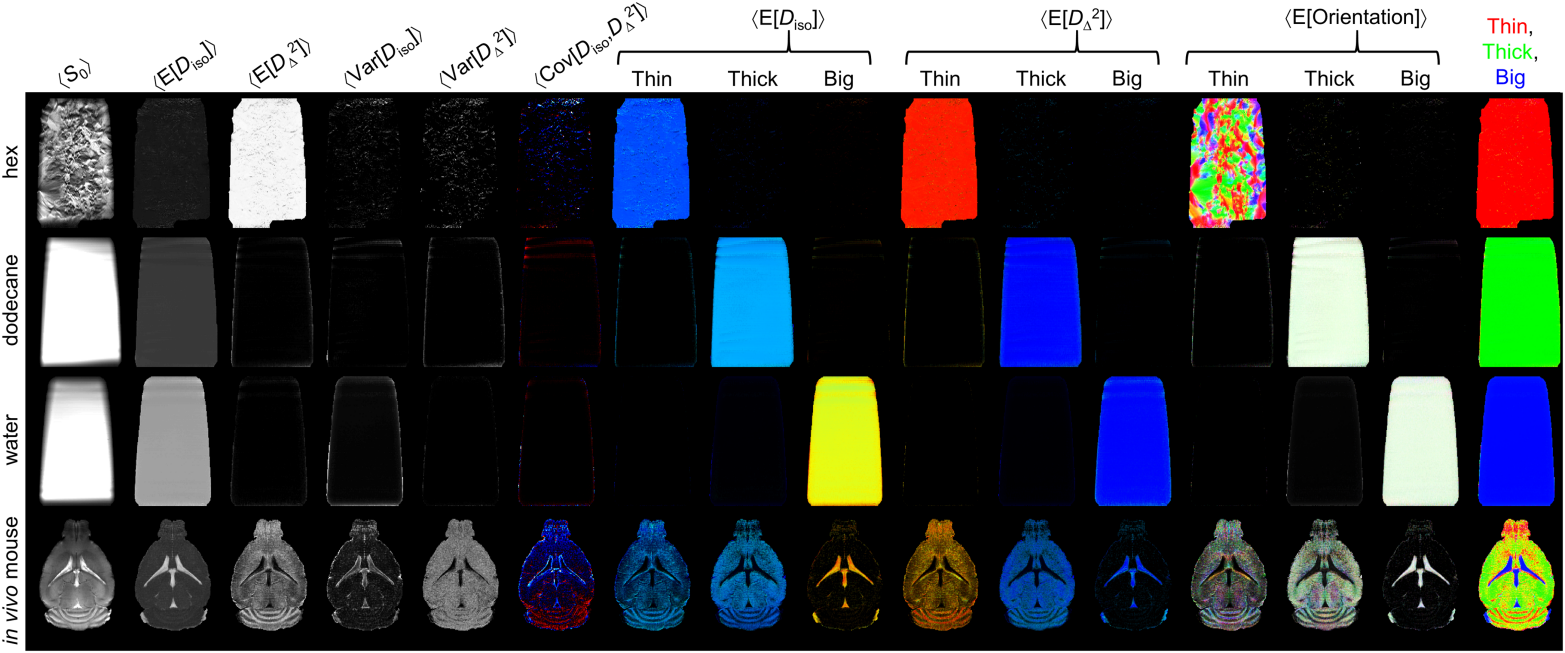
SPEN DTD parameter maps of phantoms designed to give diffusion properties similar to the white matter, gray matter and cerebrospinal fluid components in *in vivo* mouse brain. Phantom and *in vivo* data were acquired with the same hardware, imaging parameters and DTD acquisition protocol as reported in Figure 3 and Table 1. Row 1: hex^51^ (anisotropic) phantom, row 2: dodecane (slow isotropic) phantom, row 3: water (fast isotropic) phantom, row 4: *in vivo* mouse brain. The parameter maps are as explained in Figures 4 and 5. Note that for the phantoms, all heterogeneity measures Var[*D*_iso_], Var[*D*_Δ_^2^] and Cov[*D*_iso_,*D*_Δ_^2^] are zero and that all DTD components fall within the intended bins: Thin: hex phantom, Thick: dodecane, and Big: water

The borders of the three bins introduced in Figure 4 were selected to demarcate the DTD components observed in pure WM, GM and CSF voxels. In order to more thoroughly test this analysis strategy, synthetic phantoms emulating the diffusion properties of the tissue components were also investigated. This set of synthetic samples included: a “hex” phantom, where the confinement of water into nanometer‐diameter channels in a matrix of detergent and oil leads to slow anisotropic diffusion as in WM^52^; a neat liquid dodecane sample with a slow isotropic diffusion akin to that of GM^65^; and pure water, where fast isotropic diffusion happens, as in CSF. These phantoms were subjected to the same DTD SPEN analyses as the living mice brains; Figure 7 shows their results. Notice how all their DTD components fall cleanly within the designated bins: hex – “Thin”, dodecane – “Thick” and water – “Big”. For all phantoms, the 〈E[*D*_iso_]〉 and 〈E[*D*_Δ_^2^]〉 maps are also constant throughout the imaged volumes, having values consistent with expected diffusion properties. Additionally, the maps with the intra‐voxel heterogeneity measures, 〈Var[*D*_iso_]〉, 〈Var[*D*_Δ_^2^]〉 and 〈Cov[*D*_iso_,*D*_Δ_^2^]〉, show vanishingly low values. The speckled appearance of the 〈*S*_0_〉 map for the hex phantom results from variations in the local *B*_0_ field induced by the pronounced magnetic susceptibility anisotropy of the liquid crystalline domains, the orientations of which are illustrated in the 〈E[Orientation]〉 map. To facilitate comparison with the phantom data, the *in vivo* maps from Figure 6 and Figure 4 are reproduced in Figure 7. These phantom results—with their clean separation into the expected bins, constant 〈E[*D*_iso_]〉 and 〈E[*D*_Δ_^2^]〉 maps, and nearly null values of 〈Var[*D*_iso_]〉, 〈Var[*D*_Δ_^2^]〉 and 〈Cov[*D*_iso_,*D*_Δ_^2^]〉—verify that our acquisition and analysis pipeline has the capability to characterize components with the diffusion hallmarks of WM, GM and CSF. A second set of *in vivo* mouse brain DTD parameter maps and statistical descriptors is also presented in Figure S2. It has been acquired on the second mouse (*n* = 2) with identical parameters as previously described.

## 4 CONCLUSIONS

Combining the SNR advantages of very high magnetic fields and surface cryocoils with the robustness of spatiotemporal encoding towards *B*_0_‐ and *B*_1_‐inhomogeneity enabled us to image DTDs for *in vivo* mouse brains at ≈0.1 mm in‐plane spatial resolutions. As mentioned, the high in‐plane spatial resolution targeted in this study was associated with sensitivity penalties, and hence with a need for performing multi‐scan, multi‐shot interleaved averages, to obtain the kind of SNR required for nonparametric DTD inversion. SPEN MRI, which provides its data directly in image space, is well endowed to perform such joint multi‐scan/multi‐shot processing with minimal interferences from potential motional artifacts. Still, the long acquisition times required by these procedures led us to their realization in solely a single slice. Multi‐slice or 3D acquisitions would also have been possible, but keeping their overall scanning times within our self‐imposed 2.5‐hour limit would have required a substantial lowering of the spatial resolution. We estimate, for instance, that increasing the in‐plane voxel size from 100 to 170 μm would have allowed us to obtain the required SNR while reducing the per‐slice scanning time by an order of magnitude, leading to durations that would be compatible with volumetric acquisitions. This approach possessed distinct limitations including relatively long effective echo times and a limited range of *b*‐values, which may have skewed the DTDs and their derived statistical descriptors towards components with longer *T*_2_ and slower diffusivities. However, apart from a general decrease in *S*_0_ along the SPEN dimension (Figure 7), these features did not appear to introduce an evident bias in the DTD metrics, even for voxels containing multiple components with distinctly different relaxation or diffusion properties. Rigorously taking care of these effects would require including an additional acquisition dimension probing a range of echo times^63^; such investigation is onerous in terms of sensitivity and acquisition time, but it is in progress. Overall, these developments pave the way for investigating in further detail the cell densities, shapes and orientations in animal models at very high spatial resolution, thereby leading to new insights into tissue microstructure in health and disease. Further improvements of the methods presented here would enable translation into the realm of human MRI.

## FUNDING INFORMATION

The authors acknowledge support from the Israel Science Foundation (grants 2508/17 and 965/18), the Swedish Foundation for Strategic Research (AM13‐0090, ITM17‐0267), the Swedish Research Council (2018‐03697), the Kimmel Institute for Magnetic Resonance (Weizmann Institute) and the generosity of the Perlman Family Foundation.

## Supporting information


**Figure S1:** A) Map of the SNR calculated by $S_{0}/\sqrt{\mathrm{RSS}}$ with *S*_0_ the initial signal amplitude and RSS the square‐root of the residual sum of squares (RSS). B) Map of the ratio between the mean of S(*b*,*b*_Δ_ = 0) data points and their standard deviation, Mean[S(*b*,*b*_Δ_ = 0)]/SD[S(*b*,*b*_Δ_ = 0)] for *b* = 3.499·10^9^ s/m^2^. Notice that even at the maximum acquired *b*‐value some signal is remaining.
**Figure S2:** Parameter maps and statistical descriptors derived from 4D DTD acquisition of the second mouse (*n* = 2). As a reminder: total signal amplitude *S*_0_, mean E[*x*], variance Var[*x*], and covariance Cov[*x*,*y*], for the isotropic diffusivity *D*_iso_ and the squared normalized diffusion anisotropy $D_{⊗}^{2}$. The parameter maps show 〈*S*_0_〉, 〈E[*x*]〉, 〈Var[*x*]〉, and 〈Cov[*x*,*y*]〉 where the angular bracket indicate average over these 96 bootstrap realization. Per‐bin signal fractions (brightness) and per‐bin average mean values (color) are shown for the isotropic diffusivity 〈E[*D*_iso_]〉, the squared normalized diffusion anisotropy 〈E[ $D_{\Delta }^{2}$]〉, and the diffusion tensor orientation 〈E[Orientation]〉. The values of 〈E[*D*_iso_]〉 and 〈E[ $D_{\Delta }^{2}$]〉 are indicated by the corresponding linear color scales, while the 〈E[Orientation]〉 maps are color‐coded as [R,G,B] = [*D*_*xx*_,*D*_*yy*_,*D*_*zz*_]/max (*D*_*xx*_,*D*_*yy*_,*D*_*zz*_), where *D*_*ii*_ are the i‐th diagonal elements of the diffusion tensor as measured in the laboratory frame of reference.
