# Supplementary material for: Diffusion tensor distribution imaging of an in vivo mouse brain at ultrahigh magnetic field by spatiotemporal encoding
Source: NMR Biomed. 2020 Aug 19;33(11):e4355. doi: 10.1002/nbm.4355 (PMC7583469; doi:10.1002/nbm.4355)
Supplement: Supplementary file 1 — Figure S1: A) Map of the SNR calculated by S0/RSS with S 0 the initial signal amplitude and RSS the square‐root of the residual sum of squares (RSS). B) Map of the ratio between the mean of S(b,b Δ = 0) data points and their standard deviation, Mean[S(b,b Δ = 0)]/SD[S(b,b Δ = 0)] for b = 3.499·109 s/m2. Notice that even at the maximum acquired b‐value some signal is remaining. Figure S2: Parameter maps and statistical descriptors derived from 4D DTD acquisition of the second mouse (n = 2). As a reminder: total signal amplitude S 0, mean E[x], variance Var[x], and covariance Cov[x,y], for the isotropic diffusivity D iso and the squared normalized diffusion anisotropy D⊗2. The parameter maps show 〈S 0〉, 〈E[x]〉, 〈Var[x]〉, and 〈Cov[x,y]〉 where the angular bracket indicate average over these 96 bootstrap realization. Per‐bin signal fractions (brightness) and per‐bin average mean values (color) are shown for the isotropic diffusivity 〈E[D iso]〉, the squared normalized diffusion anisotropy 〈E[ DΔ2]〉, and the diffusion tensor orientation 〈E[Orientation]〉. The values of 〈E[D iso]〉 and 〈E[ DΔ2]〉 are indicated by the corresponding linear color scales, while the 〈E[Orientation]〉 maps are color‐coded as [R,G,B] = [D xx,D yy,D zz]/max (D xx,D yy,D zz), where D ii are the i‐th diagonal elements of the diffusion tensor as measured in the laboratory frame of reference. [file NBM-33-e4355-s001.pdf]

Supplementary information:

Diffusion tensor distribution imaging of an *in vivo* mouse brain at ultra-high magnetic field by spatiotemporal encoding

Maxime Yon[1*], João P. de Almeida Martins[2,3], Qingjia Bao[1], Matthew D. Budde[4], Lucio Frydman[1,*], and Daniel Topgaard[2,3]

[1]Department of Chemical and Biological Physics, Weizmann Institute, Rehovot, Israel

[2]Division of Physical Chemistry, Department of Chemistry, Lund University, Lund, Sweden

[3]Random Walk Imaging AB, Lund, Sweden

[4]Medical College of Wisconsin, Wauwatosa, USA

*lucio.frydman@weizmann.ac.il; *maxime.yon@gmail.com

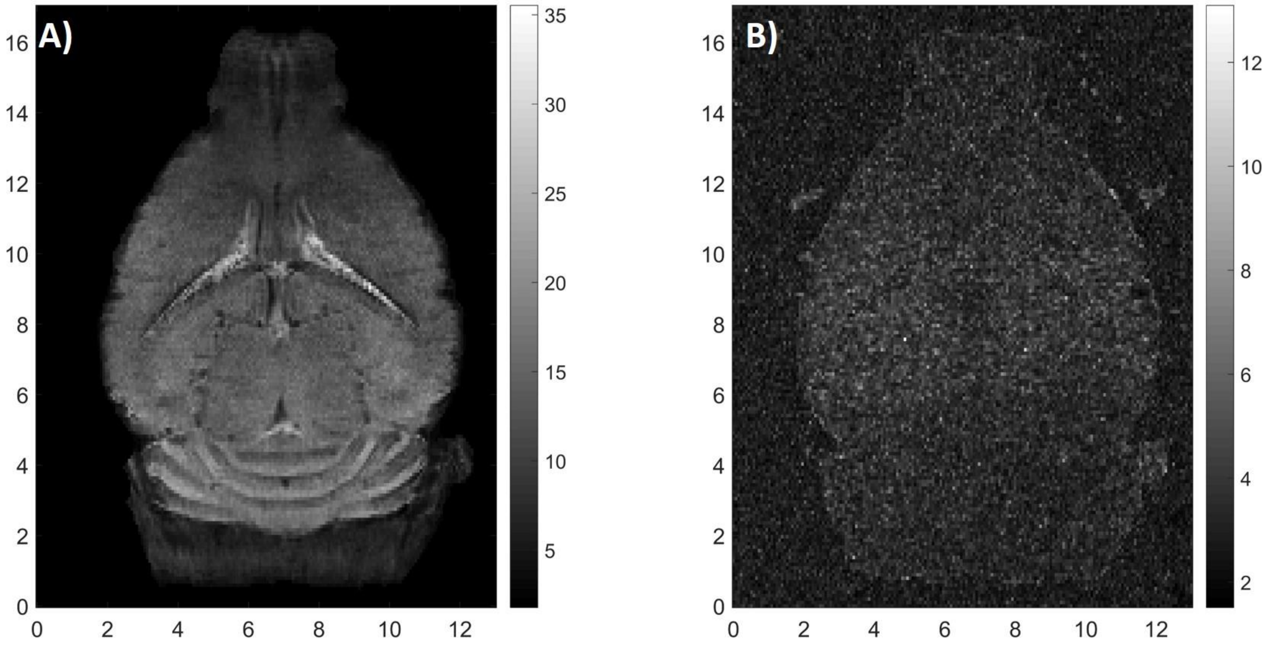

Figure S1: A) Map of the SNR calculated by $S_0/\sqrt{RSS}$ with S_0 the initial signal amplitude and RSS the square-root of the residual sum of squares (RSS). B) Map of the ratio between the mean of S(b,b_Δ=0) data points and their standard deviation, Mean[S(b,b_Δ=0)]/SD[S(b,b_Δ=0)] for b = 3.499·10^9 s/m^2. Notice that even at the maximum acquired b-value some signal is remaining.

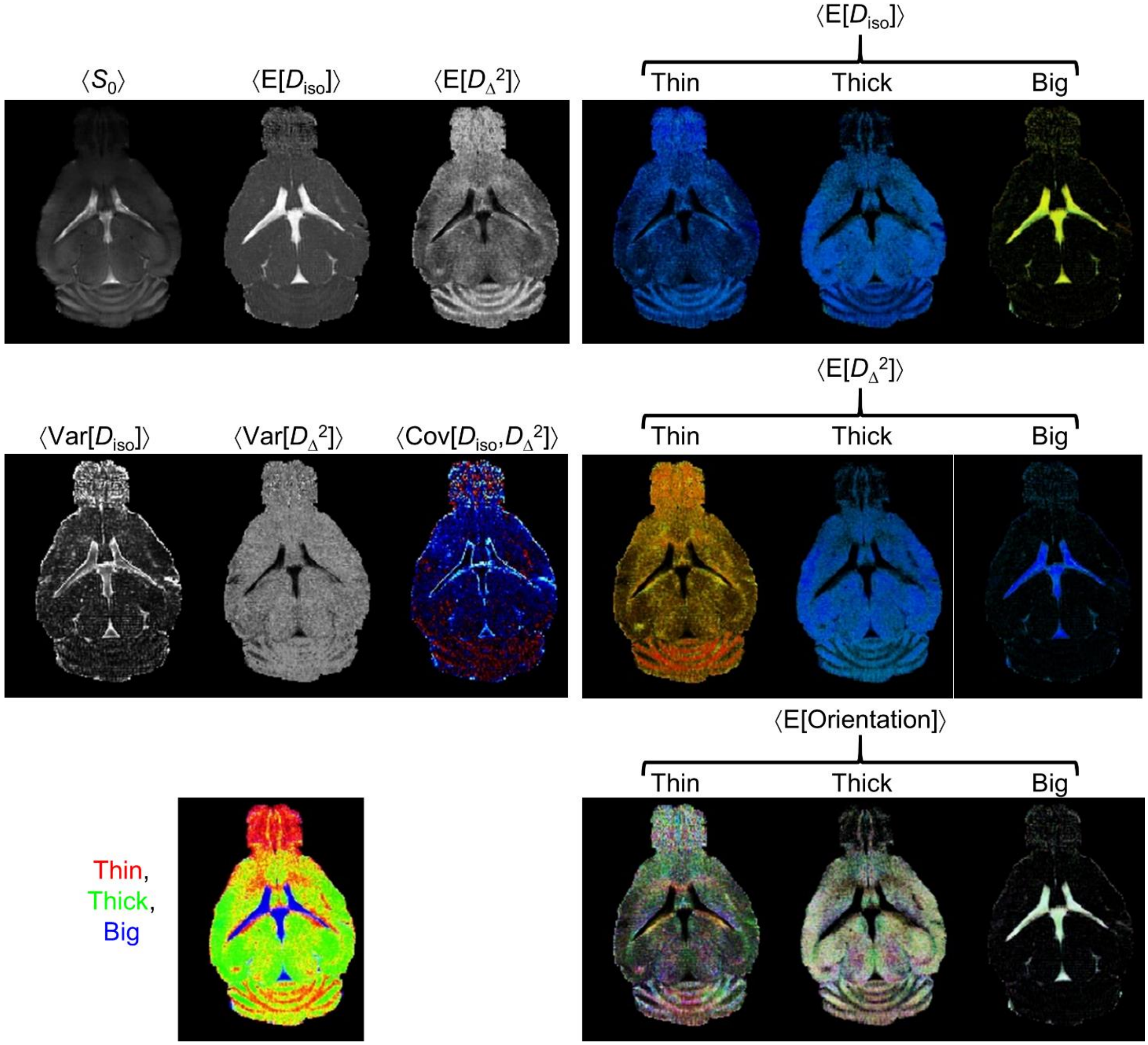

Figure S2: Parameter maps and statistical descriptors derived from 4D DTD acquisition of the second mouse (n=2). As a reminder: total signal amplitude S_0, mean E[x], variance Var[x], and covariance Cov[x,y], for the isotropic diffusivity D_{iso} and the squared normalized diffusion anisotropy $D_\Delta{}^2$. The parameter maps show ⟨S_0⟩, ⟨E[x]⟩, ⟨Var[x]⟩, and ⟨Cov[x,y]⟩ where the angular bracket indicate average over these 96 bootstrap realization. Per-bin signal fractions (brightness) and per-bin average mean values (color) are shown for the isotropic diffusivity ⟨E[D_{iso}]⟩, the squared normalized diffusion anisotropy ⟨E[$D_\Delta{}^2$]⟩, and the diffusion tensor orientation ⟨E[Orientation]⟩. The values of ⟨E[D_{iso}]⟩ and ⟨E[$D_\Delta{}^2$]⟩ are indicated by the corresponding linear color scales, while the ⟨E[Orientation]⟩ maps are color-coded as [R,G,B] = [D_{xx},D_{yy},D_{zz}]/max(D_{xx},D_{yy},D_{zz}), where D_{ii} are the i-th diagonal elements of the diffusion tensor as measured in the laboratory frame of reference.
